# Influence of Gas-Flow Conditions on the Evolution of Thermally Insulating Si_3_N_4_ Nano-Felts

**DOI:** 10.3390/ma15031068

**Published:** 2022-01-29

**Authors:** Balanand Santhosh, Mattia Biesuz, Andrea Zambotti, Gian Domenico Sorarù

**Affiliations:** Glass and Ceramics Lab, Department of Industrial Engineering, University of Trento, Via Sommarive 9, 38123 Trento, Italy; mattia.biesuz@unitn.it (M.B.); andrea.zambotti-1@unitn.it (A.Z.); giandomenico.soraru@unitn.it (G.D.S.)

**Keywords:** silicon nitride, polymer-derived ceramics, ceramic nano-belts, nitrogen flow rate, thermal insulation

## Abstract

This paper discusses the role of nitrogen (N_2_) gas flow conditions on the formation of silicon nitride (Si_3_N_4_) nano-felts from polysiloxane-impregnated polyurethane (PU) foams. The polymeric foam was converted into an amorphous silicon oxycarbide (SiOC) artefact during pyrolysis, which was then transformed, at a higher temperature, into a Si_3_N_4_ felt through a reaction between the decomposition products of SiOC with N_2_. The study identified that a N_2_ flux of ~2.60 cm.min^−1^ at the cross-section of the furnace (controlled to 100 cm^3^.min^−1^ at the inlet of the furnace using a flowmeter) substantially favored the transformation of the parent SiOC foam to Si_3_N_4_ felts. This process intensification step significantly reduced the wastage and the energy requirement while considering the material production on a bulk scale. The study also inferred that the cell sizes of the initial PU templates influenced the foam to felt transformation.

## 1. Introduction

The development of thermally and chemically stable materials for thermal insulation, especially for application at elevated temperatures, is of pivotal significance in the present economic and technical scenario. Usually, ceramics-based materials are deployed for these purposes owing to their low thermal conductivity and unrivalled stability [1]. Advanced research [2,3,4,5] is being conducted to develop ceramics-based ultra-light materials with ‘super-insulation’ properties and with very low thermal distortion. Different ceramic aerogels [6], foams [7,8], and fibrous materials [2,4] are also being studied as potential high-temperature insulants, while some are already in mass-production (www.aeropan.it, accessed on 25 January 2022), for domestic as well as industrial insulation [9].

Among the different ceramic materials in use, Si_3_N_4_ has lately gained interest in the community owing to its high thermal and mechanical stability, thermal shock resistance, and low dielectric constant and loss values [10,11,12,13,14]. Si_3_N_4_-based fibrous materials combining thermal insulation and refractory characteristics were recently proposed, also devising efficient approaches to shape the materials as required [14,15]. Among the different strategies, Si_3_N_4_ nano-felts can be obtained by the polymer-derived ceramics (PDC) route [15,16,17] using polyurethane (PU) as templates [15]. This approach reported a simple, robust, and flexible synthesis route to develop ultra-light, well-shaped fibrous Si_3_N_4_ ceramics having ultra-low thermal conductivity [15]. The controlled pyrolysis of polymeric precursor-impregnated PU-foam templates causes, at first, the formation of an amorphous SiOC foam [7,18,19], which is subsequently converted into a nano-fibrous Si_3_N_4_ artefact by reaction with N_2_ atmosphere. The evolution process of nano-felts was reported to start with the release of SiO gas (reaction (1)) followed by the reaction of this SiO gas with the C from the ceramic foam and the N_2_ gas, leading to alpha-silicon nitride (α-Si_3_N_4_) (reaction (2)) [15].

SiO_2 (s)_ + C _(s)_ → SiO _(g)_ + CO _(g)_ (1)

3SiO _(g)_ + 3C _(s)_ + 2N_2 (g)_ → Si_3_N_4 (s)_ + 3CO _(g)_ (2)

The felts were also found to have excellent chemical and thermal resistance, low thermal conductivity and mechanical performances [15], and were proposed for different applications [20,21].

The sequence of reactions (1) and (2), in order to allow the processing of an α-Si_3_N_4_ felt with optimum features and leading to a complete transformation of the parent SiOC foam, must be carefully controlled. Parameters such as the temperature and time, the cell size and density of the SiOC foam, and the flow rate of the reactive N_2_ gas may play a key role in this process. Indeed, in a previous study conducted in our lab, some parameters (temperature, time, the precursor-to-template ratio that controls the final density of the SiOC foam, and cell size) were optimized, but the gas flow conditions on the evolution were not identified and documented. In the present study, we focused our attention on the role of the flow rate on the synthesis of the Si_3_N_4_ felts. In the dynamic system in which the felt is formed, the N_2_ gas has two functions: (i) N_2_ is a reactant and needs to be available at the reaction site that is at the surface of the struts of the foam when the struts are decomposing, liberating SiO and CO gases; and (ii) N_2_ needs to act as a carrier gas to remove CO from the reaction site, allowing the decomposition to proceed further. Obviously, if the N_2_ gas is fed to the system at too slow a rate, reaction (2) may not proceed, because of the scarcity of reactants or because the CO is not removed fast enough and reaction (1) does not proceed. On the other hand, if the N_2_ flow is too high, it could remove not only CO but also SiO, before SiO could react to form the desired α-Si_3_N_4_. Hence, extended understanding and optimization of the reported fabrication process [15], considering the flow parameters, is essential to fine-tune the processing to befit the different applications envisioned.

In this work, the influence of the gas flow parameters on the evolution of Si_3_N_4_ nanobelts is reported. These studies were conducted using three starting PU templates of different cell features and at two different gas flow rates to rationalize the results obtained.

## 2. Materials and Methods

### 2.1. Materials

The commercially available polysiloxane SPR-036 (Starfire Systems Inc., Glenville, NY, USA, viscosity: 50–500 cps under room-temperature conditions) was used as the ceramic precursor without any further modifications. Platinum–divinyl tetramethyl disiloxane complex in xylene (CAS#: 68478-92-2; Sigma–Aldrich, Saint Louis, MO, USA) with a platinum content of ~2% (hydrosilylation catalyst) was used as the catalyst after diluting it to 0.1% Pt using p-xylene (CAS#: 106-42-3, Sigma–Aldrich, Saint Louis, MO, USA) to ensure its uniform dispersion in the system. Polyester-based PU foams (ARE- S.r.l, Rosate, Milan, Italy) were used as templates for fabricating the ceramic felts. Three types of PU foams having completely open cells but with different cell sizes were used for the study: PPI 45, PPI 60, and PPI 90 (PPI = pores per inch). The dimensions of the starting PU foams are given in Table S1 of ‘Supplementary Materials’. The studies were conducted with two different PU thicknesses (t): 10 mm and 20 mm, and hereafter, they are referred to as 10 and 20 for convenience, respectively.

### 2.2. Processing the ‘Nano-Felts’

**Figure 1:**
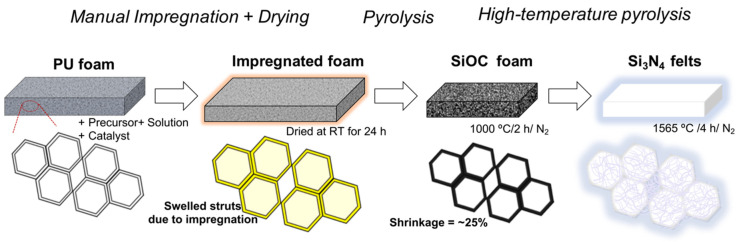
Processing of the ‘nano-felts’. The pyrolysis process that converts the impregnated polyurethane (PU) foam into the ceramic silicon oxycarbide (SiOC) foam is complete at ~1000 °C. Above that temperature, the ceramic foam is stable up to ~1500 °C and then it converts into the silicon nitride (Si_3_N_4_) felts at 1565 °C.

The processing of the felts is reported in detail in the previous works [15,21]. The samples were prepared at the previously optimized condition of 1565 °C with a holding time of 4 h, with the entire processing happening in a controlled nitrogen flow. For comparison purposes, selected samples were also pyrolyzed in Ar flow using the same temperature profile. A brief process sketch is presented in Figure 1 for a basic understanding of the process. The PU: SPR 036 weight ratio was 1:2 for all the samples. All the other main conditions were the same as what we adopted in our previous works [15,21], except for a slight modification in the flow rate of the N_2_ gas. The study was conducted at two different flow rates measured using the flowmeter:

(1) low (100 cm^3^.min^−1^), and
(2) high (600 cm^3^.min^−1^).

However, these flowrates set at the inlet of the furnace (using the flowmeter) correspond to fluxes of 2.60 cm.min^−1^ (100 cm^3^.min^−1^) and 15.58 cm.min^−1^ (600 cm^3^.min^−1^) at the cross-section of the furnace, considering the inner diameter of the alumina tube = 70 mm.

Nevertheless, henceforth, the two different flow rates are mostly referred to as 100 and 600 for convenience, respectively.

### 2.3. Material Characterizations

The bulk densities of the samples were calculated from their dimensions (measured using a Vernier caliper, sensitivity ±0.01 mm) and their weight (measured using a digital balance, sensitivity ±0.1 mg). The X-ray diffraction (XRD) spectra were collected with a D/Max- B diffractometer (Rigaku Co., Tokyo, Japan) operating at 40 kV and 30 mA with Cu Kα radiation. A digital microscope (Olympus-DSX 1000, Olympus Corp., Shinjuku City, Tokyo, Japan) was used to study the fracture surface. The scanning electron microscopy (SEM) micrographs were taken using a Supra 40 FE-SEM (Carl Zeiss NTS GmbH, Oberkochen, Germany) on the fracture surfaces after coating the samples with a thin Pt-Pd film by sputtering. The thermal diffusivities of the different felt samples were measured using the Laser Flash Analyzer, LFA 467-HyperFlash (NETZSCH-Gerätebau GmbH, Selb, Germany) in an N_2_ environment. Disk-shaped samples were made to fit in the measurement slits having a diameter = 12.7 mm, the thickness of the samples was recorded, and the parallel surfaces were coated with a thin graphite film to avoid reflection of the laser energy from the surfaces.

## 3. Results and Discussion

**Figure 2:**
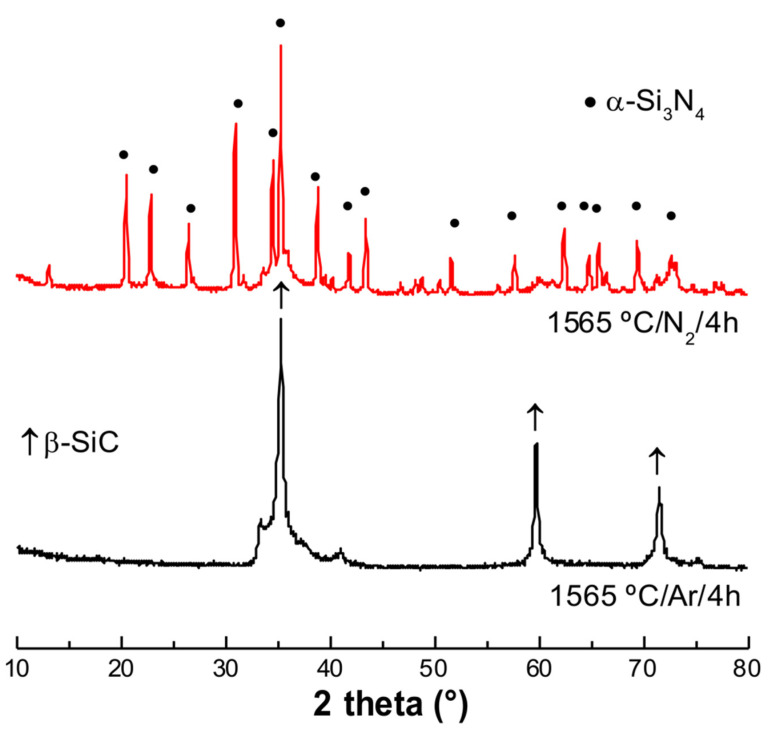
X-ray diffraction (XRD) patterns of the samples prepared in argon (Ar) and nitrogen (N_2_) atmospheres, under the same heat treatment conditions.

Pyrolytic transformation of the siloxane-impregnated PU completes at around 1000 °C [19], when a SiOC foam is formed. Silicon oxycarbides are complex amorphous materials formally consisting of a SiO_2_/SiC/C mixture. The SiOC is stable in inert atmosphere up to ca. 1400 °C. At higher temperatures, a decomposition usually occurs due to the carbothermal reduction of the silica component with the formation of SiO and CO gases [22]. In the present experiments, the temperature was increased up to 1565 °C, with a dwell for 4 h, and the XRD patterns recorded on the samples treated in N_2_ and Ar are reported in Figure 2. While pyrolysis in N_2_ results in the formation of Si_3_N_4_ (α-Si_3_N_4_ forms the primary crystalline phase; however, traces of a β-Si_3_N_4_ secondary phase can also be seen as small peaks on the diffractogram [15]), the one in Ar instead caused the formation of β-SiC (a small shoulder to the β-SiC peak at 35°, which can be seen at around 2θ = 33–35°, can be assigned to the stacking faults in β-SiC [18]) (see Figure 2).

This result can be explained considering the two-step formation process of silicon nitride as described previously: first, SiO_2_ reacts with C to form SiO_(g)_ and CO_(g)_ reaction (1); then, the reaction between SiO_(g)_, C_(s)_, and N_2(g)_ leads to the formation of silicon nitride and additional CO reaction (2). However, when the process is carried out in Ar, the second reaction cannot take place, due to the lack of N_2_ in the atmosphere. Hence, according to [23], SiO can react with carbon to form SiC + CO reaction (3):

SiO _(g)_ + 2C _(s)_ → SiC _(s)_ + CO _(g)_ (3)

**Figure 3:**
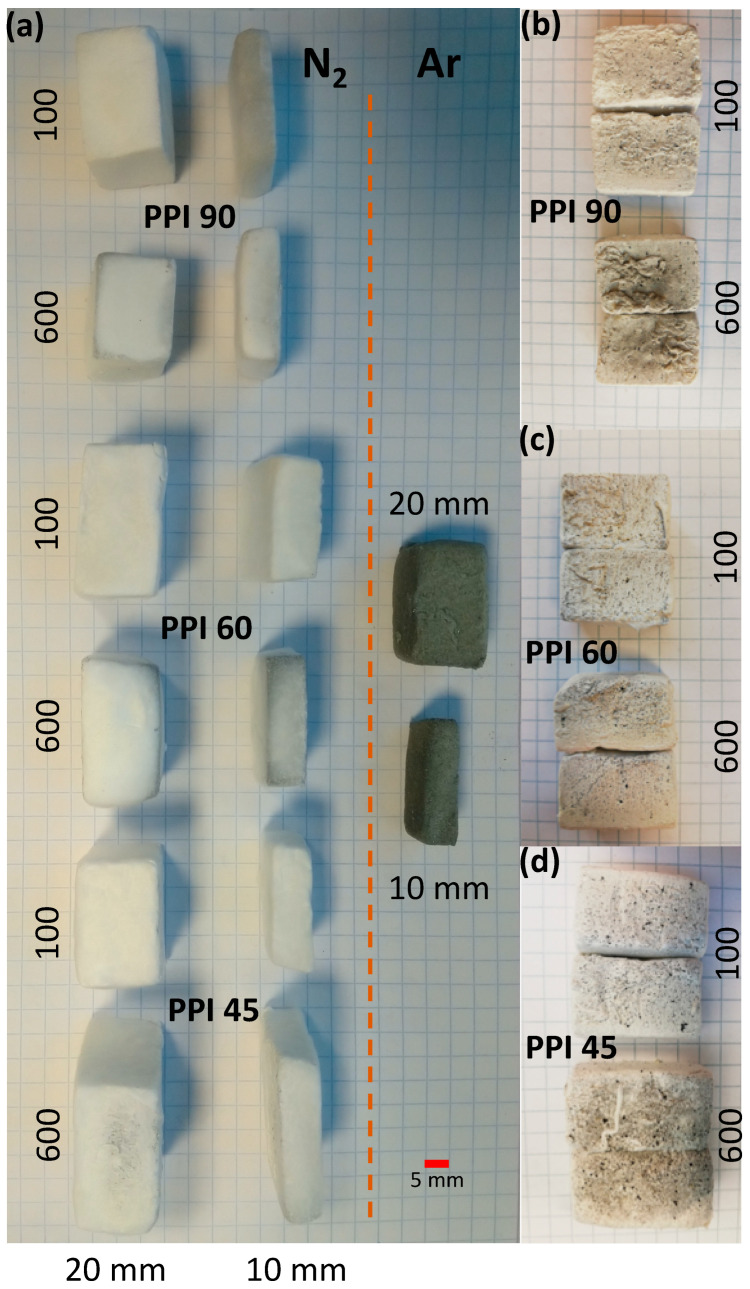
Pictures of felt samples (**a**) prepared from different PU PPIs and thicknesses, at different flow rates of N_2_ along with the control sample prepared in Ar (PPI 90, 1:2, 100 cm^3^.min^−1^), and sectioned view of the felts prepared (demonstrated using samples prepared using 20 mm PUs) at 100 cm^3^.min^−1^ and 600 cm^3^.min^−1^, (**b**) PPI 90, (**c**) PPI 60, and (**d**) PPI 45.

The density, volume shrinkage, and mass loss of the felt samples prepared at the different flow rates of N_2_ are given in Table 1. The density was found to vary in the range of 0.04–0.06 g.cm^−3^. The flow condition and the thickness of the PU templates showed only a marginal influence on the densification and the mass loss occurring. The volume shrinkage was found to increase (in general) with the thickness of the PU foams used. However, these macroscopic data were irreconcilable with the visual observation of the felts developed. It was observed that samples prepared at the highest flow rates of N_2_ (600) were found to have some darker regions, which confirm the nonuniform transformation of the foams to felts under the previously optimized conditions (see Figure 3).

**Figure 4:**
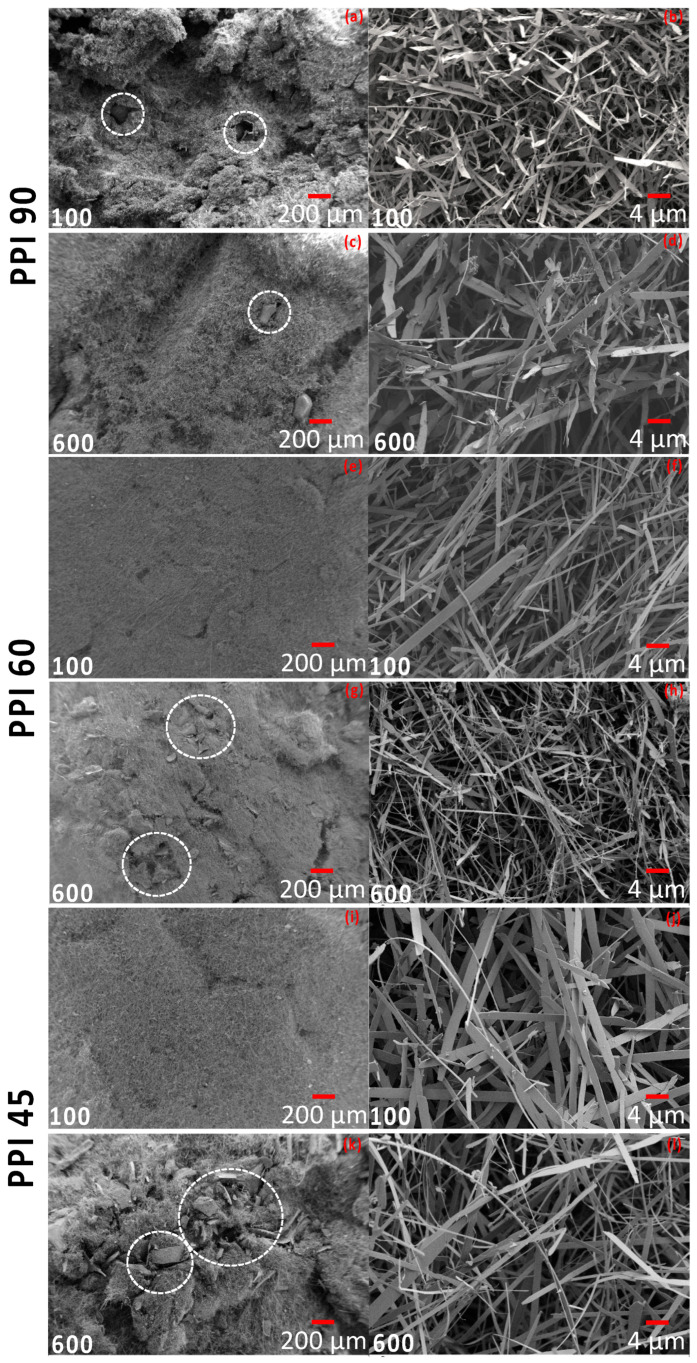
SEM micro-images of felts prepared from (**a**–**d**) PPI 90, (**e**–**h**) PPI 60, and (**i**–**l**) PPI 45 at N_2_ flow rates of 100 cm^3^.min^−1^ and 600 cm^3^.min^−1^ (images from samples prepared using 20 mm PUs; the relicts of the SiOC foams are shown in circles).

**Figure 5:**
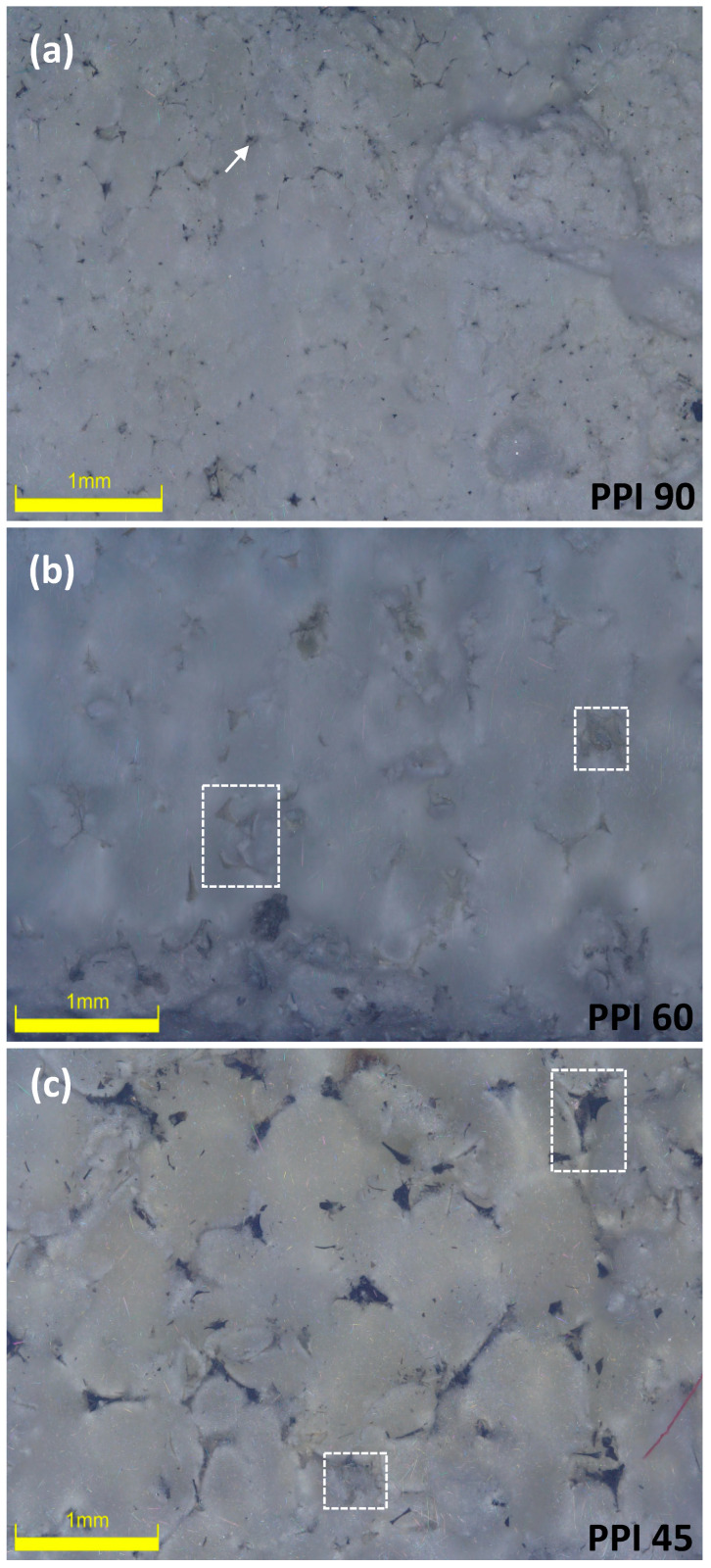
Digital microscopic images of felts prepared from (**a**) PPI 90, (**b**) PPI 60, and (**c**) PPI 45 at an N_2_ flow rate of 600 cm^3^.min^−1^ (images from samples prepared using 20 mm PUs; the relicts of the foams are shown in squares and pointed arrow).

The evolution of these nano-felts was further studied by investigating the fractured surfaces using SEM (see Figure 4). On studying the samples prepared at a higher flow rate (600 cm^3^.min^−1^), remnants of the foams were observed in the felts matrix, which were previously observed to be the dark patches on the pictures of the felts (Figure 3). This observation was clearer in the case of the samples prepared from the PUs having the larger cell-window sizes, viz. PPI 45 and PPI 60 (from the SEM images, see Figure 4). Meanwhile, the felts samples prepared from PU-PPI 90, having smaller cell-window sizes were better-transformed with less foam residues even at a flow rate of 600 cm^3^.min^−1^. Closer observations of the felts from different PPIs (on samples prepared at 600 cm^3^.min^−1^) using digital microscopes further support this observation (see Figure 5), i.e., more untransformed relicts can be seen for the samples from PPI 45 and PPI 60 than those from PPI 90. However, the samples prepared at the lower flow rates were well transformed with literally no observable relicts of the foams. These observations confirm that the PPIs and flow rates together play a role in determining the transformation degree of the foams into Si_3_N_4_ felts. We can rationalize the experimental results as follows. The high flow rates of N_2_ increase the rate of removal of SiO_(g)_, formed through reaction (1), from the reaction site where it should combine, through reaction (2), with N_2_ and C to form Si_3_N_4_, reducing, accordingly, the transformation yield. Similarly, the smaller cell size (as in PPI 90) helps retain the SiO better than the larger cell sizes, as PPI 45 and PPI 60 (see Figure 5). In summary, preventing SiO from diffusing out of the foam, either by decreasing the N_2_ flow rate or decreasing the cell size of the starting PU foam, before it can react to form Si_3_N_4_, helps in obtaining a better transformed felt.

**Figure 6:**
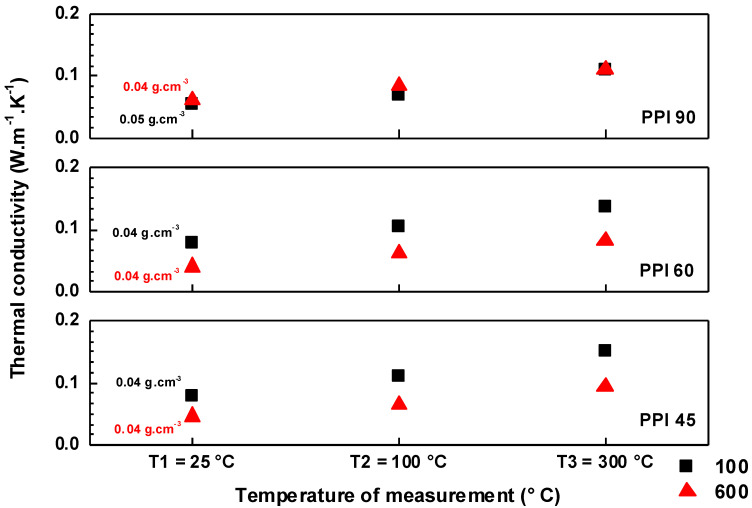
Thermal conductivity of the felts prepared from PPI 45, PPI 60, and PPI 90. The thermal diffusivity was measured in an N_2_ environment on the felts prepared from 10 mm PUs, and the measurements were taken at RT (25 °C), 100 °C, and 300 °C.

The thermal diffusivity (*κ*) of the felts was measured at three temperatures, viz. RT, 100 °C, and 300 °C. The thermal conductivity (*λ*) was calculated using the specific heat capacity (*Cp*) data from [15], *κ*, and the bulk density (*ρ*), following the equation λ=ρ∗Cp∗κ [18], and the results are presented in Figure 6. In the case of the felts prepared using PPI 45 and PPI 60, the *λ* computed for the felts prepared at 600 cm^3^.min^−1^ (flow rate of N_2_) was lower than that of the ones prepared at 100 cm^3^.min^−1^. However, in the case of PPI 90, the *λ* calculated showed only marginal variation. The presence of a low-conducting phase in the felts (the unconverted foam relicts that appear as dark regions (see Figure 3 and Figure 4)), prepared at a higher flow rate, could lower the total λ by forming an extra interface in the conducting matrix. However, more investigation is needed to explain this observation.

The dark relicts can be seen in all the felts samples from all three PPIs (45, 60, and 90) at a higher flow rate (600 cm^3^.min^−1^) (see Figure 3); however, the influence was more protuberant in the case of the lower PPIs (45 and 60). The microscopic and SEM observation agrees with this remark (see Figure 4 and Figure 5); the transformation in PPI 90 is better despite the different flow conditions (N_2_ flow rate) (i.e., a lesser amount of relicts remained in the case of felts from PPI 90 at a 600 cm^3^.min^−1^ flow in comparison to PPI 45 and PPI 60 at 600 cm^3^.min^−1^).

## 4. Conclusions

The influences of N_2_ gas and the gas flow rates on the evolution and transformation of ultra-light Si_3_N_4_ nano-belts, synthesized via a PDC approach, by the controlled pyrolysis of polysiloxane-impregnated PU foams, were testified. The samples were made following the previously optimized and reported preparation conditions: 1565 °C/4 h/N_2_-atmosphere. The study was conducted using two different flow rates; 100 cm^3^.min^−1^ (low-flow) and 600 cm^3^.min^−1^ (high-flow) evidenced that the low flow rates (100 cm^3^.min^−1^, which corresponds to a N_2_ flux of ~2.60 cm.min^−1^ at the cross-section of the furnace) favored a better transformation of the foam → felt. The high flow of N_2_ gas washed away the reactive gases such as SiO, formed during the process, which were vital for the nanobelt formations. The smaller cell sizes of the PU templates (PPI 90) also favored the nano-felts transformation even at higher gas flow rates compared to the ones with bigger cell sizes used in the study (PPI 45 and 60).

## Figures and Tables

**Table 1:** The bulk density, volumetric shrinkage, and mass loss (%) of nano-felts samples prepared with different PPIs, having varied thicknesses, and under different N_2_ flow conditions.

PU Foam	PU Foam Thickness (mm)	N_2_-Flow Conditions (cm^3^.min^−1^)	Bulk Density (g.cm^−3^)	Volume Shrinkage (%)	Mass Loss (%)
PPI 90	10	100	0.05	55.3	67.2
	20	100	0.06	52.5	64.4
	10	600	0.04	51.6	68.0
	20	600	0.04	53.5	69.3
PPI 60	10	100	0.04	42.0	72.4
	20	100	0.04	48.1	70.7
	10	600	0.04	49.1	67.6
	20	600	0.05	49.6	65.4
PPI 45	10	100	0.04	47.6	69.2
	20	100	0.04	51.4	70.8
	10	600	0.04	41.7	70.9
	20	600	0.05	47.9	66.0

## Supplementary Materials

The following supporting information can be downloaded at: https://www.mdpi.com/article/10.3390/ma15031068/s1, Figure S1: Pictures of felt samples prepared from different PU PPIs at a N_2_ flow rate of 300 cm^3^.min^−1^: (a) top view, and (b) side view (demonstrated using samples prepared using 10 mm PUs with a SPR 036: PU (by wt.) = 2:1); Table S1: The dimensions of the starting PU foams; Table S2: The bulk density, volumetric shrinkage, and mass loss (%) of nano-felts samples prepared with different PPIs at flow rates 100 cm^3^.min^−1^, 300 cm^3^.min^−1^, and 600 cm^3^.min^−1^ having varied thickness.
