# Supplementary material for: Influence of Gas-Flow Conditions on the Evolution of Thermally Insulating Si3N4 Nano-Felts
Source: Materials (Basel). 2022 Jan 29;15(3):1068. doi: 10.3390/ma15031068 (PMC8839865; doi:10.3390/ma15031068)
Supplement: Supplementary file 1 [file materials-15-01068-s001.zip › materials-1535810-supplementary.pdf]

materials

MDPI

Supplementary Material

Influence of Gas-Flow Conditions on the Evolution of Thermally Insulating Si_3N_4 Nano-Felts

Balanand Santhosh *, Mattia Biesuz, Andrea Zambotti and Gian Domenico Sorarù

Glass and Ceramics Lab, Department of Industrial Engineering, University of Trento, Via Sommarive 9, 38123 Trento, Italy; mattia.biesuz@unitn.it (M.B.); andrea.zambotti-1@unitn.it (A.Z.); giandomenico.soraru@unitn.it (G.D.S.)

* Correspondence: balanand.santhosh@unitn.it

Table S1. The dimensions of the starting PU foams.

PU foam	N_2-flow conditions ($cm^3.min^{-1}$)	Dimensions (mm)		
		Length (l)	Width (w)	Thickness (t)
PPI 90	100	30	31	10
	100	27	30	20
	600	30	31	10
	600	27	29	20
PPI 60	100	29	30	10
	100	30	36	20
	600	35	32	10
	600	35	30	20
PPI 45	100	34	25	10
	100	32	30	20
	600	33	37	10
	600	37	38	20

Table S2. The bulk density, volumetric shrinkage, and mass loss (%) of nano-felts samples prepared with different PPIs at flow rates 100 $cm^3.min^{-1}$, **300 $cm^3.min^{-1}$**, and 600 $cm^3.min^{-1}$ having varied thickness.

PU foam	Foam dimension (thickness)	N_2-Flow conditions ($cm^3.min^{-1}$)	Density ($g.cm^{-3}$)	Volume shrinkage (%)	Actual mass loss (%)
PPI 90	10 mm	100	0.05	55.3	67.2
	20 mm	100	0.06	52.5	64.4
	**10 mm**	**300**	**0.04**	**48**	**70.3**
	**20 mm**	**300**	**0.05**	**50**	**60.8**
	10 mm	600	0.04	51.6	68.0
	20 mm	600	0.04	53.5	69.3
PPI 60	10 mm	100	0.037	42	72.4
	20 mm	100	0.04	48.1	70.7
	**10 mm**	**300**	**0.04**	**51**	**-**
	20 mm	300	-	-	-
	10 mm	600	0.044	49.1	67.6
	20 mm	600	0.048	49.6	65.4
PPI 45	10 mm	100	0.04	47.6	69.2
	20 mm	100	0.04	51.3	70.8
	**10 mm**	**300**	**0.03**	**53**	**75.7**
	**20 mm**	**300**	**0.05**	**52**	**64.9**
	10 mm	600	0.04	41.7	70.9
	20 mm	600	0.05	47.9	66.0

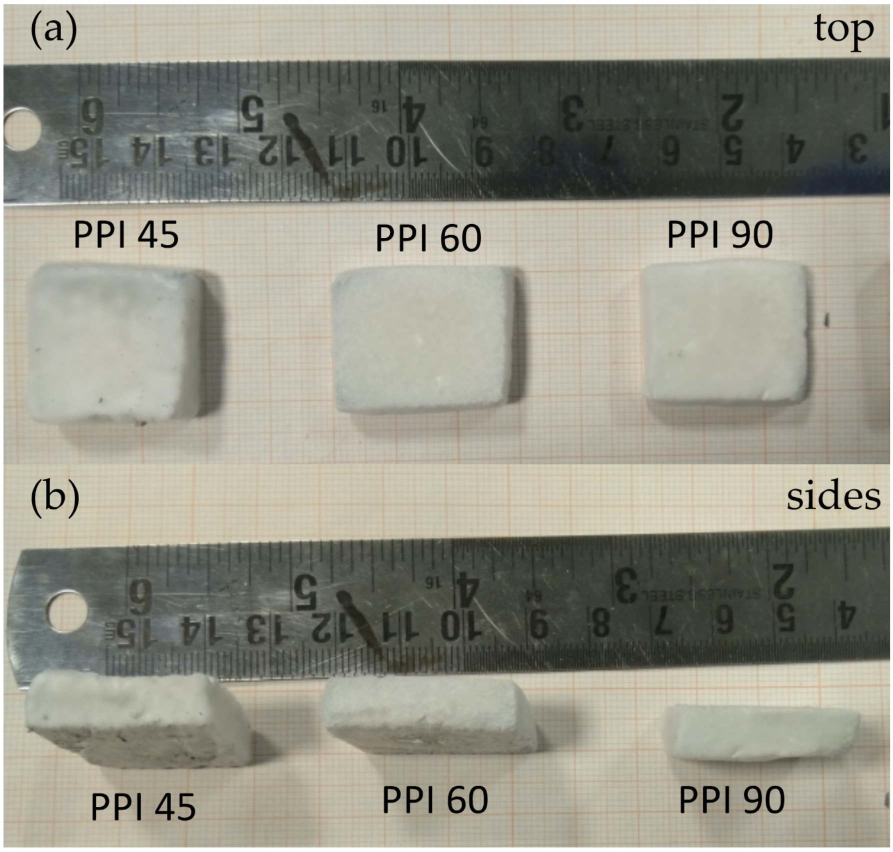

Figure S1. Pictures of felt samples prepared from different PU PPIs at a N_2 flow rate of **300 cm^3.min^{-1}**: (a) top view, and (b) side view (demonstrated using samples prepared using 10 mm PUs with a SPR 036: PU (by wt.) = 2:1).
